# Topological phase transition and robust pseudospin interface states induced by angular perturbation in 2D topological photonic crystals

**DOI:** 10.1038/s41598-023-27868-x

**Published:** 2023-01-16

**Authors:** Daniel Borges-Silva, Carlos H. Costa, Claudionor G. Bezerra

**Affiliations:** 1 Departamento de Física, Universidade Federal do Rio Grande do Norte, Natal, RN 59078-970 Brazil; 2 Instituto Federal do Ceará, Cedro, CE 63400-000 Brazil; 3 LAREB, Universidade Federal do Ceará, Russas, CE 62900-000 Brazil

**Keywords:** Photonic crystals, Topological defects

## Abstract

In recent years the research about topological photonic structures has been a very attractive topic in nanoscience from both a basic science and a technological point of view. In this work we propose a two-dimensional topological photonic structure, composed of a trivial and a topological photonic crystals, made of dumbbell-shaped dielectric rods. The topological behavior is induced by introducing an angular perturbation in the dumbbell-shaped dielectric rods. We show that this composed structure supports pseudospin interface states at the interface between the trivial and topological crystals. Our numerical results show that a bandgap is opened in the band structure by introducing the angular perturbation in the system, lifting the double degeneracy of the double Dirac cone at the $\Gamma$ point of the Brillouin zone, despite keeping the $C_6$ symmetry group. A pseudospin topological behavior was observed and analyzed with emphasis on the photonic bands at the $\mathbf{\Gamma }$ point. We have also investigated the robustness of these pseudospin interface states and, according with our numerical results, we conclude that they are robust against defects, disorder and reflection. Finally, we have shown that the two edge modes present energy flux propagating in opposite directions, which is the photonic analogue of the quantum spin Hall effect.

## Introduction

The study of the topological photonic crystals is one of the most attractive topics in condensed matter in the last years. This is a consequence of their incredible capacity to simulate electronic phenomena, such as quantum Hall effect^1–3^, quantum valley Hall effect^4–12^, quantum spin Hall effect^13–18^ and topological insulators^19–21^. The topological behavior of two-dimensional photonic crystals is classified by the corresponding Chern number^22,23^. For example, a zero Chern number corresponds to a trivial photonic system, while a non-zero Chern number corresponds to a topological one. The Chern number is defined for each photonic band, but this topological invariant may also be defined, for a given bandgap, as the sum of the Chern numbers of all bands below that specific bandgap^24^. It is possible to induce a topological phase in two-dimensional photonic crystals by introducing perturbations in the system, so that a mass term is added in the effective Hamiltonian and a non-trivial bandgap emerges^25^. That non-trivial topological bandgap can support robust edge states, which are topologically protected against defects, disorder and reflection at the interfaces^26–28^.

It is known from the literature that photonic crystals in a triangular lattice, with $C_6$ point symmetry group, exhibit a double Dirac cone at the $\mathbf {\Gamma }$ point of the Brillouin zone^16,29^, and the degenerated bands present *p*- and *d*-waves orbitals in the band structure associated to the transverse magnetic (TM) polarization of the electric field^13,31^. On the other hand, we can lift this double degeneracy by perturbing the system while keeping the $C_6$ symmetry group. As a consequence, a complete bandgap is opened in the band structure^34^ and a topological phase is induced in the system. For example, in photonic structures on a honeycomb lattice composed of dielectric cuboids^35^. Thus, edge states will emerge in the non-trivial bandgap, which are robust against disorder and defects because of the bulk-edge correspondence and topological protection^36–38^. Therefore, the propagating modes can travel in well-defined engineered directions. Edge modes presenting those features are excellent candidates for technological applications once the flux of light can be controlled without any significant energetic loss or reflection.

In this work we propose a two-dimensional topological photonic crystal with a very specific geometry: it is composed of dumbbell-shaped dielectric rods in a triangular lattice with six rods in the unit cell. We will show later that we can induce a complete bandgap in the system, by introducing an angular perturbation in the rods orientation, having as a consequence the emergence of a topological band gap. The physical system addressed here is of current interest for a large audience because of its potential technological applications. For example, topological photonic crystals may be applied as waveguides, optical filters and may enhance the efficiency of optical fibers. Also, from a basic science point of view, photonic crystals presents photonic analogues of physical phenomena such as quantum Hall effect and quantum spin Hall effect. This work is organized as follows. In Sect. “Photonic crystal” we introduce the system and describe its features. In Sect. “Topological phase transition” we introduce the angular perturbation and investigate the topological behavior of the system. In Sect. “Robust pseudospin interface states” we study the emergence of edge states around the interface between the topological and trivial photonic crystals. We also address the robustness of the interface states against defects and disorder. Finally, in Sect. “Conclusions” we summarize the main results obtained in this work.

## Photonic crystal

**Figure 1:**
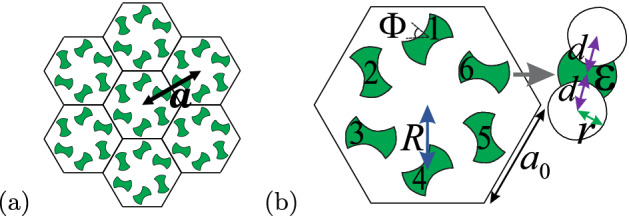
Unperturbed 2D Photonic Crystal. (**a**) Scheme of the triangular lattice with six dumbbell-shaped rods. (**b**) Scheme of the unit cell of the unperturbed dumbbell-shaped rod photonic crystal. Here $a_0=a/\sqrt{3}$, $R=a/3$ is the distance from the center of the unit cell to the center of the dumbbell-shaped rod, $r=0.13a$, $d=R/2$ and $\Phi$ is the angular orientation of each rod.

The photonic structure considered here is a triangular photonic crystal of six“artificial atoms” composed by dumbbell-shaped dielectric rods ($\varepsilon =11.7$), surrounded by air. The construction of the dumbbell-shaped rods is as follows. We consider three cylindrical rods of radius $r=0.13a$, with $a=1$ μm being the lattice constant. Then, two of them are shifted by $d=\pm 0.5R$ from the center of the reference cylindrical rod. Here $R=a/3$ is the distance between the unit cell center and the center of the reference cylindrical rod. Afterward, we consider the region of the reference cylindrical rod which is not superimposed with the other two shifted cylinders as the dumbbell-shaped dielectric rod (see Fig. 1).

The six dumbbell-shaped rods in the unit cell can be rotated around their respective centers by the orientation angle $\Phi$, as illustrated in Fig. 1. We can observe from Fig. 1 that the angular orientation of the rods allows different configurations for the unit cell. Thus, we can change their angular orientation by introducing an angular perturbation, which may open a bandgap at the $\Gamma$ point. Therefore, we expect that this angular perturbation will lead to a topological phase transition from trivial to non-trivial domain^35,39^. The angular perturbation is introduced in the orientation angle $\Phi$ of the rods, so that we can write the orientation angle of the *i*-th dumbbell-shaped rod as

1 $$\begin{aligned} \Phi _i=(i-1){\pi \over 3}+\phi _0+\phi . \end{aligned}$$

**Figure 2:**
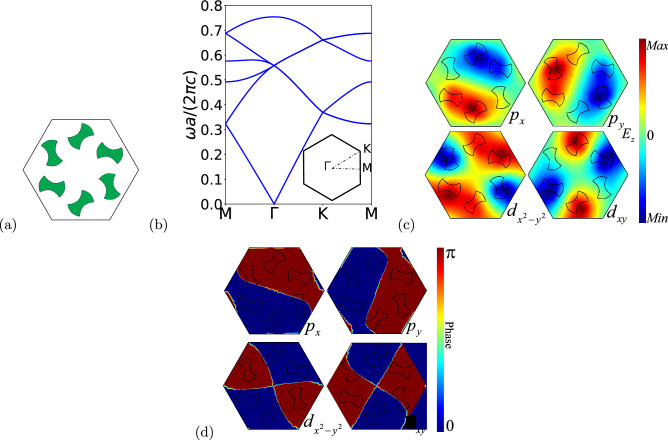
Results for the unperturbed photonic crystal. (**a**) Scheme of the unit cell with angular perturbation $\phi =0$. (**b**) Band structure for the TM modes with $\varepsilon =11.7$, $a=1 \mu m$, $R=a/3$, $r=0.13a$, $d=R/2$ and $\phi =0$. (**c**) Profile of the electric field $E_z$ at the Dirac point. We can see that pseudospin states are dipole modes (upper panels) and quadrupole modes (lower panels). (**d**) Phase of the electric field $E_z$ at the Dirac point. We can see that pseudospin states are dipole modes (upper panels) and quadrupole modes (lower panels). (We used COMSOL Multiphysics v.6.0. www.comsol.com. sofware to create the image).

Here, $i=1,2,...,6$ is the rod index, $\phi _0$ is the initial unperturbed angle and $\phi$ is the angular perturbation introduced in our system. Because of the symmetry of the dumbbell-shaped rod, $0<\phi <\pi$. Here we assume $\phi _0=\pi /4$ (see Fig. 2a). We should remark that the initial unperturbed angle $\phi _0=\pi /4$ is chosen so that we obtain the band structure without bandgap (see Fig. 2b). This is because $\phi _0=\pi /4$ corresponds to a tight-binding model with uniform hopping in the same lattice (for details see Ref.^35^).

It has been reported in the literature that triangular lattices, with 6 artificial atoms in the unit cell, have two irreducible representations in the $C_6$ point symmetry group, which are associated with the symmetry of the lattice^40^. This point symmetry group allows the occurrence of a double degenerate Dirac cone at the center of the Brillouin zone^41,42^. Moreover, degenerated bands related to the double degeneracy present pseudospin behavior, which is associated to dipole and quadrupole modes, i.e., $p_x$ ($p_y$) and $d_{xy} (d_{x^2-y^2}$) orbitals corresponding to odd (even) parity in the real space, respectively^13^. That irreducible representation allows us to write the pseudospin states as^43–45^,

2 $$\begin{aligned} p_{\pm }=\frac{1}{\sqrt{2}}\left( p_x\pm ip_y\right) , \quad  d_{\pm }=\frac{1}{\sqrt{2}}(d_{x^2-y^2}\pm id_{xy}). \end{aligned}$$

The band structure of the system was obtained through the software COMSOL Multiphysics^46^, which is based in the finite element method (FEM). The TM modes ($E_z,H_x,H_y\ne 0$), for the unperturbed case $\phi =0$, are shown in Fig. 2b. We can observe from Fig. 2b, due to the $C_6$ symmetry group, a double degenerated Dirac point at $\mathbf{\Gamma }$ point of the Brillouin zone. In Fig. 2c and d we plot the profile of the electric field along the *z* direction ($E_z$) and the phase of the electric field, respectively, at the double degenerate Dirac point with $\omega a/(2\pi c)=0.5575$. The pseudospin sates that are related to dipole modes correspond to $p_x$ and $p_y$ orbitals, while the pseudospin sates that are related to quadrupole modes correspond to $d_{xy}$ and $d_{x^2-y^2}$ orbitals. We can observe from Fig. 2d that the electric field presents two well defined phases, 0 and $\pi$, with an abrupt transition between them. In the next section we will show the procedure to lift the double degeneracy of those states and obtain a complete photonic bandgap.

The magnetic field associated to pseudospin up and down states is given by the Faraday relation. For example, for the *p* orbital we have $\mathbf {H}_{\pm }=-(i/\mu _0\omega )\nabla \times [(p_x\pm ip_y)\hat{z}]$. Since the $p_x$ and $p_y$ modes are connected to each other by a $\pi /2$ rotation, we can write the magnetic field as (for more details see the supplemental material of Ref.^13^),

3 $$\begin{aligned} \mathbf {H}_{\pm }=(H_{p_x,x}\pm iH_{p_x,y})(\hat{x}{\mp } i\hat{y}). \end{aligned}$$

**Figure 3:**
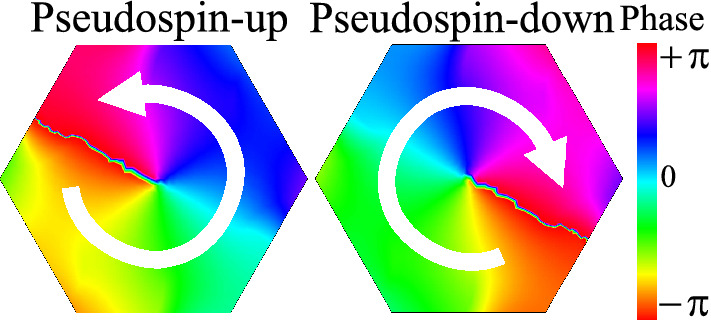
Circular polarization of the magnetic field related to the pseudospin modes ($H_{\pm }$). Notice that psedusospin up presents an anticlockwise circular polarization, while pseudsospin down presents a clockwise circular polarization. (We used COMSOL Multiphysics v.6.0. www.comsol.com. sofware to create the image).

The term $i\hat{y}$ corresponds to a ${\mp } \pi /2$ phase shift of the *x* and *y* components of the magnetic field, leading to a circular polarization. This circular polarization corresponds to the angular momentum of the electric field. The pseudospin state can be identified according to the circular polarization of the magnetic field, e.g., an anticlockwise circular polarization is associated to the pseudospin up and a clockwise circular polarization is associated to the peudospin down^47^. This is illustrated in Fig. 3, i.e., the pseudospin up is represented by an anticlockwise circular polarization of the magnetic field, while a pseudospin down is represented by a clockwise circular polarization of the magnetic field.

## Topological phase transition

**Figure 4:**
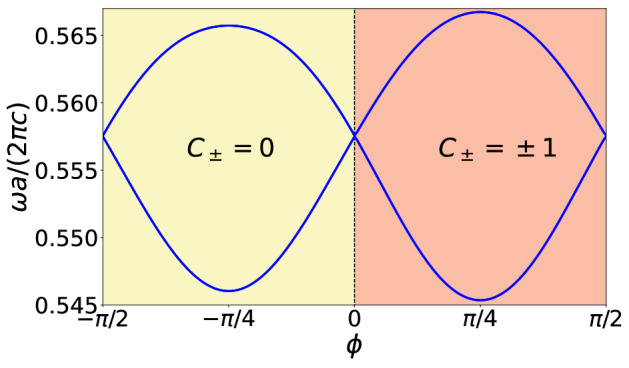
Effect of the perturbation in the band structure. Upper and lower edges of the bandgap as a function of $\phi$, at the $\mathbf{\Gamma }$ point, with $R=a/3$, $r=0.13a$, $d=R/2$, and $\varepsilon =11.7$. For $\phi =0$ and $|\phi |=\pi /2$, the bands are doubly degenerate resulting in a Dirac cone. For $\phi \ne 0$ and $|\phi |\ne \pi /2$ a bandgap is opened. (We used COMSOL Multiphysics v.6.0. www.comsol.com. sofware to create the image).

In order to lift the double Dirac cone and open a complete photonic bandgap, we induce an angular perturbation on the range $-\pi /2\le \phi \le \pi /2$ at the $\mathbf{\Gamma }$ point, as it is shown in Fig. 4. It should be observed that for the unperturbed case ($\phi =0$) we have four degenerate bands resulting in the double degenerate Dirac cone. On the other hand, for the perturbed case $\phi \ne 0$, the perturbation lift the double degeneracy and a bandgap is opened, despite keeping the $C_6$ symmetry group. We can observe from Fig. 4 that as $|\phi |$ increases from 0, the bandgap width monotonically increases, reaching its maximum at $|\phi |=\pi /4$. On the contrary, as $|\phi |$ increases from $\pi /4$, the bandgap width monotonically decreases, until the double Dirac cone is recovered at $|\phi |=\pi /2$. This is a consequence, as mentioned before, that the perturbation has a period of $\pi$.

It is clear from Fig. 4 that $\phi$ opens a bandgap at the $\mathbf{\Gamma }$ point for both positive and negative angular perturbation. However, those bandgaps have different topological behavior. Therefore, we need to investigate their topological invariant, the Chern number, for a better understanding of their bulk and edge modes. Thus, in order to study the topological behavior close to the $\mathbf{\Gamma }$ point, we write an effective Hamiltonian around it by using the $\mathbf {k}$.$\mathbf {p}$ perturbed model. The Chern number of the bandgap is then given by^48–49^,

4 $$\begin{aligned} C_{\pm }=\pm \frac{1}{2}[\text {sign}(B)+\text {sign}(M)]. \end{aligned}$$

**Figure 5:**
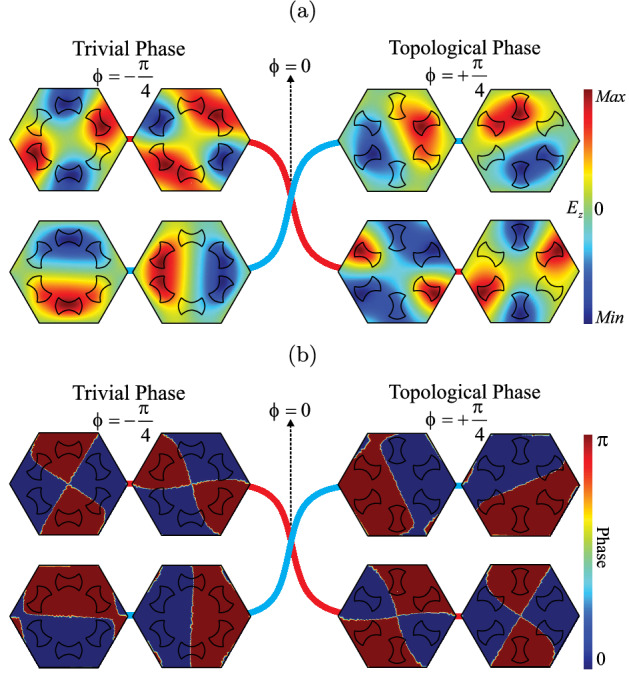
Topological phase transition diagram. Band inversion between $\phi =-\pi /4$ and $\phi =\pi /4$. (**a**) Electric field $E_z$. For $-\pi /4 \le \phi <0$ the frequency of the dipole modes is lower than the frequency of the quadrupole modes, corresponding to the trivial case (left panels). For $0<\phi \le \pi /4$ the frequency of the dipole modes is higher than the frequency of the quadrupole modes, corresponding to the topological case (right panels). (**b**) Phase of the electric field $E_z$. We can observe that the topological phase transition is also characterized by a phase shift of $\pi$ in the electric field. (We used COMSOL Multiphysics v.6.0. www.comsol.com. sofware to create the image).

Here *B* is the diagonal term of the effective Hamiltonian close to the $\mathbf{\Gamma }$ point, which is obtained from second-order correction and is essentially negative^13^. Also, $M=(\varepsilon _d-\varepsilon _p)/2$, where $\varepsilon _d$ and $\varepsilon _p$ are the eigenmodes of orbit *d* and orbit *p*, respectively^16^. The eigenmode $\varepsilon _p$ is related to the doubly degenerate dipole states of $p_{\pm }$, while $\varepsilon _d$ is related to the doubly degenerate quadrupole states of $d_{\pm }$^48^. If $\varepsilon _p<\varepsilon _d$ then $M>0$, hence $C_{\pm }=0$ and the photonic crystal is topologically trivial. However, if $\varepsilon _p>\varepsilon _d$ then $M<0$ and $C_{\pm }=\pm 1$ and the photonic crystal is in a topological phase. So, the inversion of the bands between the degenerated modes at the $\mathbf{\Gamma }$ point promotes the topological phase transition^31^. In fact, the inversion of the bands takes place at $\phi =0$ as we can see in Fig. 5. The phase with $\phi <0$ corresponds to $C_{\pm }=0$, while the phase with $\phi >0$ corresponds to $C_{\pm }=\pm 1$ (see Fig. 4).

Let us illustrate the inversion of the bands and its relation with the topological phase. We consider the inversion of the bands that takes place between $\phi =-\pi /4$ and $\phi =\pi /4$ for the degenerated bands, as shown in Fig. 5. It is possible to notice from Fig. 5a that for the negative perturbation case ($\phi <0$) the frequency of the dipole modes is lower than the frequency of the quadrupole modes, while for the positive perturbation case ($\phi >0$) the frequency of the dipole modes is higher than the frequency of the quadrupole modes. Therefore, for $-\pi /4 \le \phi <0$ we obtain $C_{\pm }=0$ that corresponds to a trivial photonic crystal. On the other hand, for the case $0<\phi \le \pi /4$ we obtain $C_{\pm }=\pm 1$ that corresponds to a topological photonic crystal, as expected. In addition to that, Fig. 5b shows the phase of the electric field for the cases of $\phi =-\pi /4$ and $\phi =\pi /4$. It is possible to notice a phase shift of $\pi$, when the topological phase transition takes place, in agreement with the literature^34^.

**Figure 6:**
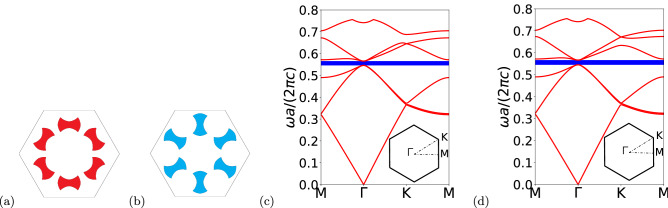
Results for the perturbed photonic crystal. Scheme of the unit cell with (**a**) $\phi =-\pi /4$ and (**b**) $\phi =\pi /4$. (**c**) Band structure for the TM modes with $\varepsilon =11.7$, $a=1 \mu m$, $R=a/3$, $r=0.13a$ and $d=R/2$, for the trivial case ($\phi =-\pi /4$). (**d**) The same for the topological case ($\phi =\pi /4$). The bandgap is highlighted by the shaded area.

The band structures for the trivial and topological photonic crystals, with angular perturbation $\phi \ne 0$, are shown in Fig. 6. The perturbation opens a gap between $\omega a/(2\pi c)=0.5462$ and $\omega a/(2\pi c)=0.5657$, for the trivial photonic crystal case, and between $\omega a/(2\pi c)=0.5453$ and $\omega a/(2\pi c)=0.5667$, for the topological photonic crystal case, corresponding to gap-midgap ratios^50^ of $\Delta \omega /\omega _m=0.0350$ and $\Delta \omega /\omega _m=0.0385$, respectively. Once the bandgap is opened, edge states can appear inside the gap and these states may present very interesting features as we will see in the next section.

## Robust pseudospin interface states

**Figure 7:**
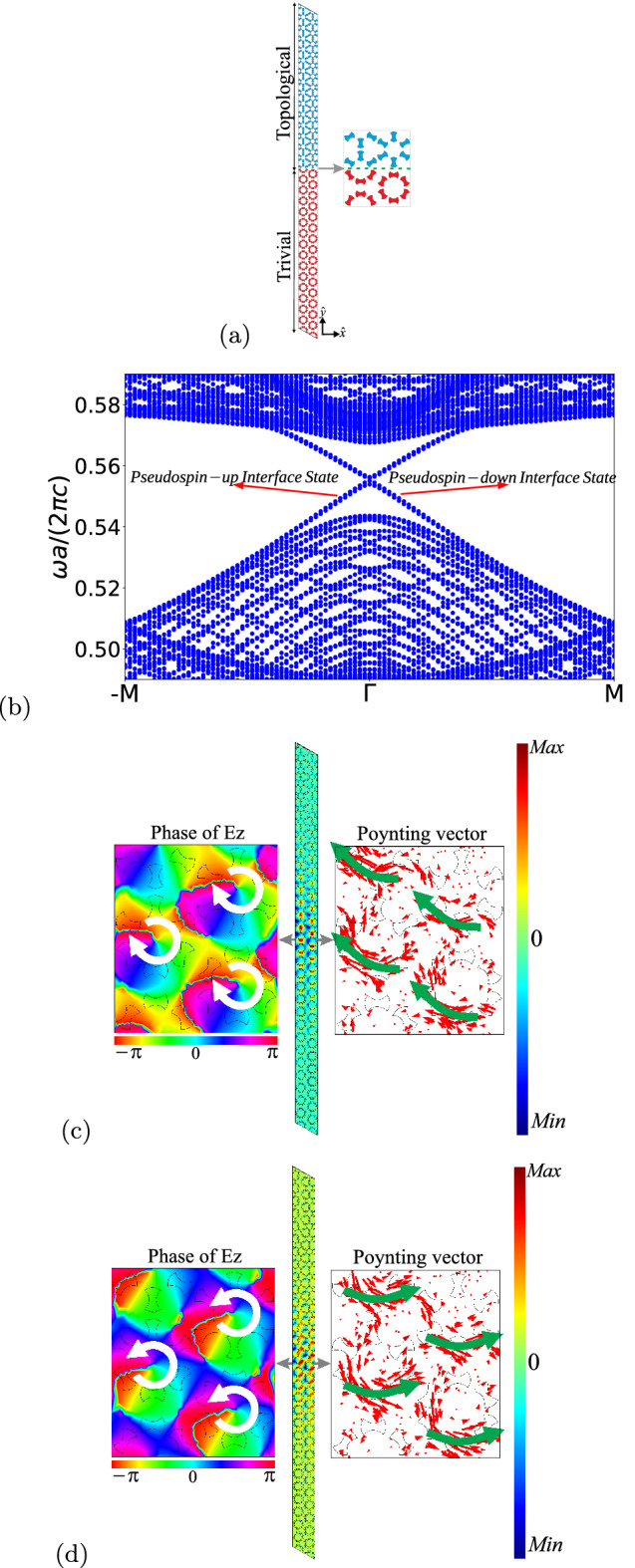
Results for a sample composed of topological and trivial photonic crystals. (**a**) Schematic of the supercell with 15 trivial unit cells ($\phi =-\pi /4$) and 15 topological unit cells ($\phi =\pi /4$) making an interface. (**b**) Projected band structure along the $\Gamma \rightarrow M$ direction for TM modes. We can observe two interface modes in the gap. (**c**) Spacial distribution of $E_z$, the time-averaged Poynting vector (right), and phase distribution (left), for $\omega a/(2\pi c)=0.5532$ at $k_x=0.015$ $k_0$. (**d**) The same for for $\omega a/(2\pi c)=0.5596$ at $k_x=0.015$ $k_0$. (We used COMSOL Multiphysics v.6.0. www.comsol.com. sofware to create the image).

The perturbed system presents both the topological and trivial bandgaps associated to the angular perturbation $\phi$ of the dumbbell-shaped rods. It is known from the literature that the edge-bulk correspondence guarantees that if we build a slab composed of two photonic crystals, with different topological invariants, i.e., Chern numbers, robust edge modes, localized around the interfaces between the photonic crystals, emerge^45,51–53^. Once the edge modes are localized at the interfaces, the corresponding edge states are called interface states. The interface states are topologically protected and are robust against defects, disorder and allow transmission without any reflection, with no significant energetic loss^28,54,55^. In order to investigate the emergence of the interface states in our system, we build a supercell with 15 trivial unit cells ($\phi =-\pi /4$) and 15 topological unit cells ($\phi =\pi /4$), with an interface between them, and then we calculate the projected band structure along the $\mathbf{\Gamma \rightarrow M}$ direction, as we can see in Fig. 7.

It is possible to observe two topological interface modes inside the gap, which present a mirror symmetry around the $\mathbf{\Gamma }$ point, i.e. $\omega a/(2\pi c)(-\mathbf {k})=-\omega a/(2\pi c)(\mathbf {k})$. From Fig. 7b we can observe that they cross each other right at the $\mathbf{\Gamma }$ point, which means that the two topological interface modes are degenerated at the $\mathbf{\Gamma }$ point. Moreover, taking into account the group velocity $\mathbf {v}_g=\mathbf {\nabla }_k \omega$, we conclude that those modes are traveling in different directions. In Fig. 7c and d are shown the spacial distributions of $E_z$, along the supercell, the time-averaged Poynting vector ($\mathbf {S}=Re[\mathbf {E}\times \mathbf {B}^*]/2$), and the phase of $E_z$, around the interface, for $\omega a/(2\pi c)=0.5532$ and $\omega a/(2\pi c)=0.5596$, respectively, with $\mathbf {k}=k_x\hat{x}=0.015$ $k_0\hat{x}$ and $k_0=4\pi /(\sqrt{3}a)$. It is easy to observe that the modes are localized around the interface, so that the intensity of the electric field is reduced in the bulk (we will quantify this behavior later). Furthermore, by comparing their Poynting vectors, it is clear that the interface states present energy flux in different directions (see the arrows in Figs. 7c and d). Moreover, we can identify that the mode with $\omega a/(2\pi c)=0.5532$ presents a clockwise polarization, while the mode with $\omega a/(2\pi c)=0.5596$ presents an anticlockwise polarization, which correspond to a pseudsopin down and up, respectively. So, we can associate a pseudospin number to each interface mode ($\pm 1/2$) according to the propagation direction and energy flux^56^. The pseudospin-up ($+1/2$) is associated to the interface state with group velocity and energy flux from the left to the right, while the pseudospin-down ($-1/2$) is associated to the interface state with group velocity and energy flux from the right to the left^14^ (see Fig. 7b). Fig. 7c and Fig. 7d show the spatial distribution of $E_z$ for pseudospin-down and -up, respectively. The electromagnetic energy propagates at the interface in opposite directions according to those two pseudospins states ($\pm 1/2$). This behavior is analogous to the quantum spin Hall effect (QSHE). Therefore, we may conclude that the structure investigated here presents the *photonic spin Hall effect* (PSHE)^57–60^.

**Figure 8:**
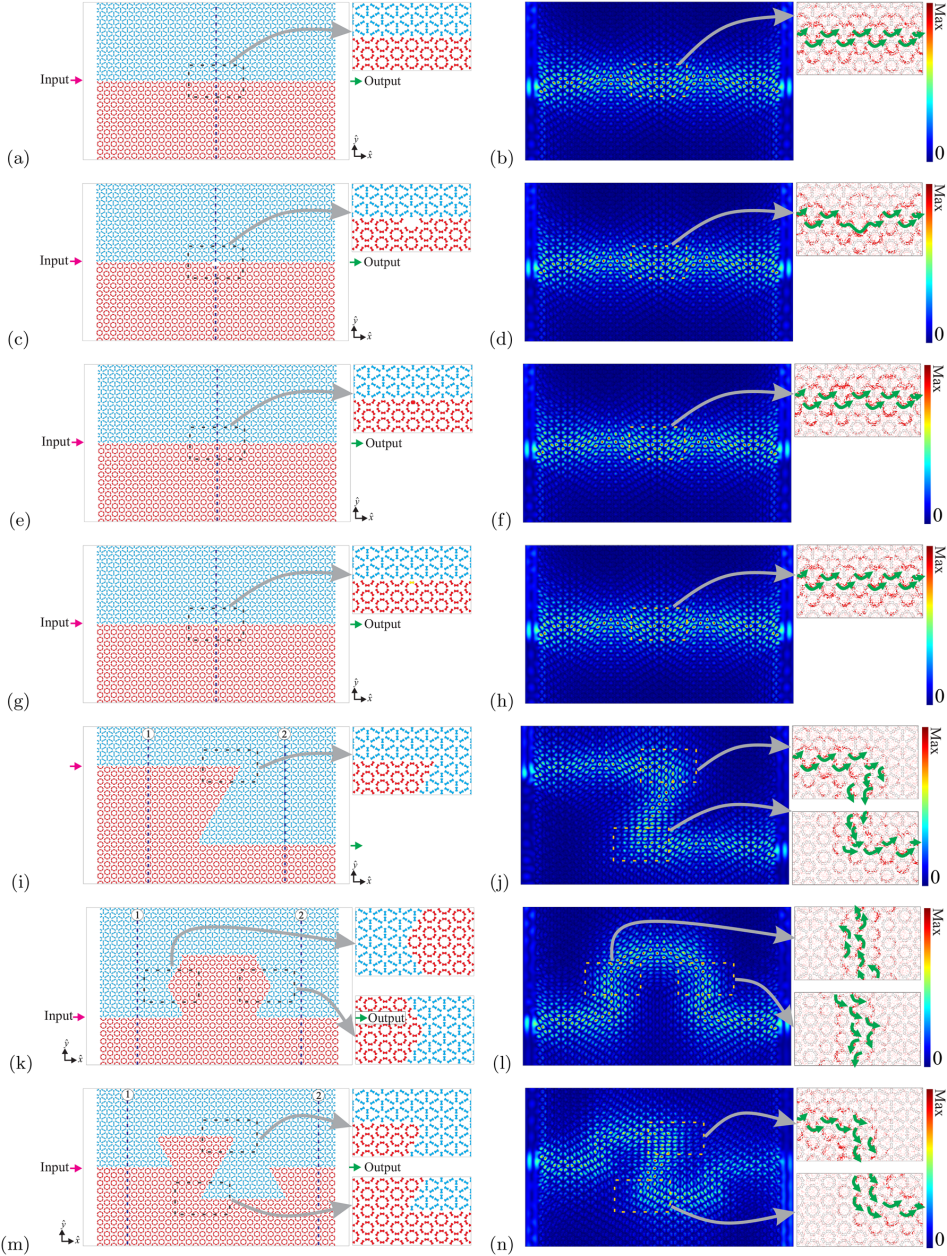
Simulation of the propagation of light along the photonic structure. Schematic for (**a**) interface between trivial and topological photonic crystals without defect, (**c**) interface with cavity defect, (**e**) interface with a larger rod, (**g**) interface with an Al rod, (**i**) Z interface, (**k**) Omega interface and (**m**) double horseshoe interface. The source of light is on the left and a detector is on the right. In all cases $\phi =-\pi /4$ and $\phi =\pi /4$, respectively. Spatial distribution of the normalised electric field $E_N$ with $\omega a/(2\pi c)=0.5462$ for (**b**) interface without defect, (**d**) interface with cavity defect, (**f**) interface with a larger rod, (**h**) interface with an Al rod, (**j**) Z interface, (**l**) Omega interface and (**n**) double horseshoe interface. The Poynting vector directions are illustrated by the arrows. (We used COMSOL Multiphysics v.6.0. www.comsol.com. sofware to create the image).

Let us now investigate the robustness of the pseudospin interface states against disorder and defects, as well as, their localization at the interface^61–64^. To do so, we build a $(25,20\sqrt{3}/2)a$ slab with an interface between the trivial ($\phi =-\pi /4$) and topological ($\phi =\pi /4$) photonic crystals. We consider a source of propagating waves by using a“port on” on the left and a detector by using a“port off” on the right, as it is depicted in Fig. 8a. Then, we plot the normalized electric field profile defined as $E_N=\sqrt{|E_z|^2}$ for $\omega a/(2\pi c)=0.5462$, as it is shown in Fig. 8b. We also considered different defects at the interface: (i) a cavity by removing a rod (see Fig. 8c), (ii) a larger dumbbell-shaped rod of size $d_0=0.6R$ (the bigger one in Fig. 8e), (iii) an Al dumbbell-shaped rod (see Fig. 8g), (iv) a Z interface (see Fig. 8i), (v) an Omega interface (see Fig. 8k) and (vi) a double horseshoe interface (see Fig. 8m). The normalized electric field profiles $E_N$ corresponding to these defects, for $\omega a/(2\pi c)=0.5462$, are shown in Figs. 8d, f, h, j, l, and n, respectively. Besides the normalized electric field profiles, the time-averaged Poynting vectors are also illustrated in Fig. 8 (see the arrows in the zoom windows). We can observe from Fig. b, d, f, h and j, that the electric field survives and the energy flux remains unchanged along the interface for all defects considered here. Notice that the Poynting vectors present small changes only around the defects or around the corners of the interfaces, but their global behavior and direction (see the big arrows) remain unchanged for all defects considered here.

It is important to realize from Fig. 7a that the supercell present an interface composed of a complete unit cell alternating with a broken unit cell. The results obtained from this interface are completely analogous to the results obtained from an interface purely composed of a complete unit cell, or an interface purely composed of a broken unit cell. Thus, the interface states are going to emerge inside the bandgap and these states present a pseudospin behavior no matter is the interface’s shape. In fact, this is an expected result, once the emergence of the interface modes is fully guaranteed by the Bulk-Edge correspondence. It means that the only request for the emergence of the edge modes around the interface is the nonzero difference between Chern numbers from the crystals that compose the supercell in Fig. 7a and the sample in Fig. 8.

**Figure 9:**
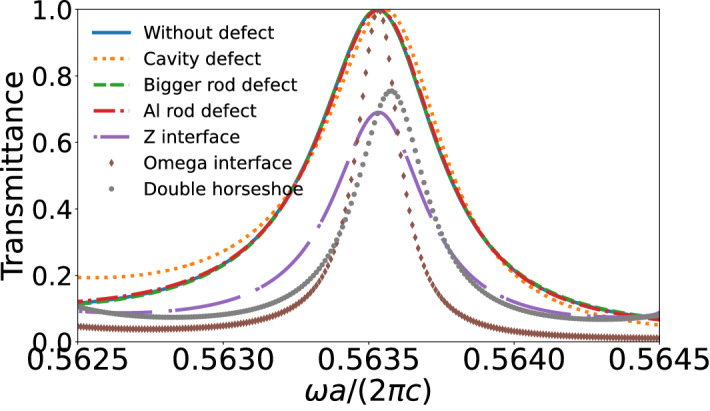
Transmittance spectrum for the cases without defect, cavity defect, larger rod defect, Al rod defect, Z interface, Omega interface and double horseshoe interface.

The qualitative information from Fig. 8 is corroborated by the quantitative information provided by the transmittance of the system^22,65,66^. In order to confirm that statement, the transmittance between “port on”and “port off”,corresponding to the slabs schematized in Fig. 8, are plotted in Fig. 9. We can observe from Fig. 9 that the transmittance peak practically does not change for the case without defect, cavity defect, larger rod defect and Al rod defect. We could expect some losses when we introduce the Al defect, once it is made by a metallic material, but those losses are much smaller than the transmittance and we can neglect them. On the other hand, for the Omega interface, Z interface and double horseshoe interface, the transmittance peak goes around 1.0, 0.7 and 0.8, respectively. Those cases present different behaviors because they are built by bending the orientation of parts of the original interface. Note that for: (i) the Z interface case light faces two direction changes; (ii) the Omega interface case light faces six direction changes; and (iii) the double horseshoe interface case light faces six direction changes with larger angles. Therefore, once those interface cases are not small and localized defects, it is expected that its transmittance be a little different from the previous cases. As an overall conclusion, our results show with no doubt that the transmittance peaks survive even in the presence of the complex defects considered here. Thus, it is shown that the pseudospin interface states are robust against defects and disorders introduced into the system.

**Figure 10:**
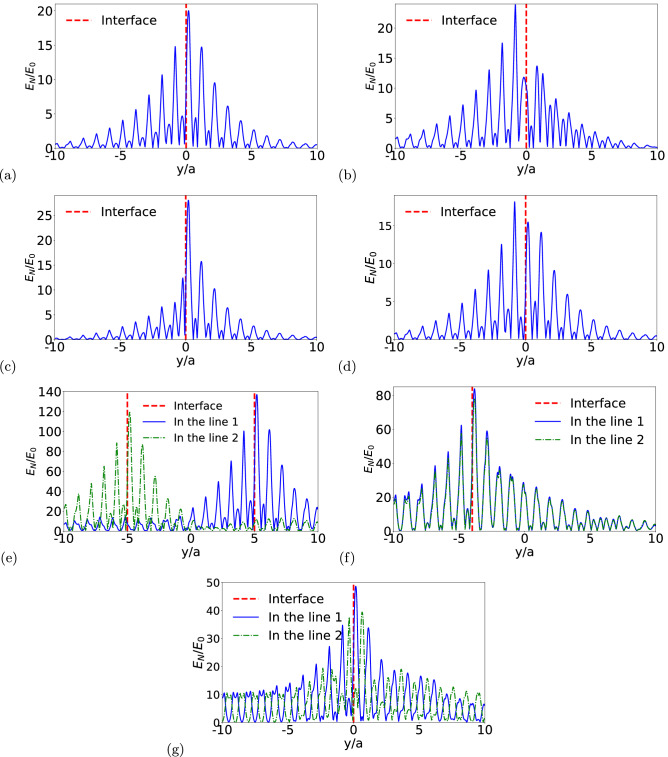
Localization of the interface states. Intensity of $E_N/E_0$ versus *y* with $\omega a/(\pi c)=0.5462$ for (**a**) interface without defect, (**b**) small cavity defect, (**c**) larger rod defect, (**d**) Al rod defect, (**e**) Z interface, (**f**) Omega interface and (**g**) double horseshoe interface.

Before concluding, let us analyze from a quantitative point of view the localization of the pseudospin states around the interface. This localization was illustrated from a qualitative point of view in Fig. 8. In order to quantify the localization, we calculate $E_N/E_0$ point-by-point on a line perpendicular to the interface (see Fig. 8). Here $E_0=k_e e/a^2$, where $k_e$ is the Coulomb constant, *a* is the lattice constant and *e* is the modulus of the elementary electric charge. Figs. 10a–g show the intensity of $E_N/E_0$, for $\omega a/(\pi c)=0.5462$, taking into account the interface: (i) without defect, (ii) with cavity defect, (iii) with a larger rod, (iv) with an Al rod, (v) with a Z interface, (vi) with an Omega interface and (vii) with a double horseshoe interface, respectively. Analyzing Fig. 10 it is possible to observe that the electric field is localized around the interfaces, inclusive in the presence of defects and also for the cases of the Z interface, the Omega interface and the double horseshoe interface. The electric field in the bulk, far from the interface, is very low in both the topological and trivial photonic crystals. In particular, for the cases of the Z interface, the Omega interface and the double horseshoe interface, we plot $E_N/E_0$, also for $\omega a/(\pi c)=0.5462$, on two lines: (i) line 1 located before the corners of the interface and (ii) line 2 located after the corners of the interface. Those calculations are illustrated in Fig. 10e–g. Once again it is confirmed the localization of the electric field around the interface, no matter in which part of the interface the calculation is performed. Therefore, we can conclude that the system proposed in this work presents a typical topological insulator behavior, i.e., it supports robust pseudospin states, allowing propagation at the border (interface), but with no propagation in the bulk^67–70^.

## Conclusions

In summary, we have proposed a 2D topological photonic system, composed of dumbbell-shaped dielectric rods in a triangular lattice with six rods in the unit cell. The band structure of the system was obtained through the software COMSOL Multiphysics^46^, which is based in the finite element method (FEM). We have found that a complete bandgap is opened in the system by introducing an angular perturbation $\phi$ in the rods orientation. As a consequence of the perturbation, two interface states emerge between the topological and trivial photonic crystal, which are localized around the interface and topologically protected. Our results show that the interface states present energy flux propagating in opposite directions. In addition to that, they have opposite winding of the electromagnetic field’s phase and Poynting vector, so that we can associate a pseudospin number to each interface mode $(\pm 1/2)$, now called pseudospin interface states according to the rotation direction of Poynting vector. This behavior is analogous to the quantum spin Hall effect (QSHE). Accordingly, the structure investigated here presents the *photonic spin Hall effect* (PSHE). Furthermore, topological protection makes the pseudospin interface states robust against defects and disorder. This is well illustrated trough the calculation of the transmittance of the system for: (i) a cavity defect, (ii) a larger rod defect, (iii) an Al rod defect, (iv) a Z interface, (v) an Omega interface and (vi) a double horseshoe interface. For all defect cases considered here the transmittance is mildly affected, which means that the system proposed is an excellent candidate for technological applications, once the flux of light can be controlled without any significant energetic loss or reflection. Finally, from the calculation of the electric field intensity far from the interface, we can conclude that the system proposed in this work presents a typical topological insulator behavior, i.e. it supports robust pseudospin states, allowing propagation at the border (interface), but with no propagation in the bulk. As a concluding remark, it is worth to emphasize that the angular perturbation introduced in this work has lifted the double degeneracy, of the double Dirac cone at the $\Gamma$ point of the Brillouin zone, despite keeping the $C_6$ symmetry group. Certainly the 2D topological photonic system proposed in this work can be realized experimentally, and we hope that experimentalists are encouraged to investigate it.

## Data Availability

The datasets generated during and/or analysed during the current study are available from the corresponding author on reasonable request.
